# The Impact of a Smartphone App on the Quality of Pediatric Colonoscopy Preparations: Randomized Controlled Trial

**DOI:** 10.2196/18174

**Published:** 2020-11-10

**Authors:** James Brief, Anupama Chawla, Diana Lerner, Bernadette Vitola, Robert Woroniecki, Jeffrey Morganstern

**Affiliations:** 1 Department of Pediatric Gastroenterology Stony Brook Children’s Hospital Stony Brook, NY United States; 2 Department of Pediatric Gastroenterology Medical College of Wisconsin Milwaukee, WI United States; 3 Department of Pediatric Nephrology Stony Brook Children’s Hospital Stony Brook, NY United States

**Keywords:** colonoscopy, app, pediatrics, prep, smartphone, mobile phone, mHealth

## Abstract

**Background:**

Smartphone apps have been successfully used to help adults prepare for colonoscopies. However, no study to date has investigated the effect of a smartphone app on pediatric colonoscopy preparation.

**Objective:**

The aim of this study is to determine if an app (*SB Colonoscopy Prep*) designed to educate and guide patients through their colonoscopy preparation will yield benefits over paper-based instructions and information.

**Methods:**

In total, 46 patients aged 5-18 years received either app-based or written material with instructions on how to take their prep medications as well as information about the colonoscopy procedure. Prep quality, the number of calls to the gastroenterology service, and patient arrival time were recorded. After the procedure, a questionnaire was given to each patient through which they graded their knowledge of the procedure both before and after receiving the app or written material.

**Results:**

App users had higher mean Boston scores versus control subjects receiving written instructions (7.2 vs 5.9, *P*=.02), indicating better colonoscopy preps. In total, 75% (15/20) of app users and 41% (9/22) of written instruction users had preps categorized as “excellent” on the Boston scale. We found no significant differences in knowledge about the procedure (app users: 10/20 [50%], written instruction users 8/22 [36%]; *P*=.37), phone calls to the gastroenterology clinic (n=6 vs n=2; *P*=.27), or arrival times at the endoscopy suite (44 min vs 46 min before the scheduled procedure time; *P*=.56).

**Conclusions:**

Smartphone app use was associated with an increased number of colonoscopy preps classified as “excellent” on the Boston scale. There was no significant difference between app users and the control group regarding the number of calls to the gastroenterology clinic, patient arrival time, or patient knowledge about the procedure.

**Trial Registration:**

ClinicalTrials.gov NCT04590105; https://clinicaltrials.gov/ct2/show/NCT04590105

## Introduction

Pediatric colonoscopy is integral for diagnosis and treatment of a number of pediatric gastrointestinal conditions including hematochezia, inflammatory bowel disease, and colonic polyps.

Up to one-third of children undergoing these procedures are reported to have suboptimal colon preparation [1]. Poor-quality preparation can increase the risk and length of the procedure, the rate of missed diagnosis, and the cost of the procedure if it is repeated or rescheduled. Studies in adult patients from Pillai et al [2], Hayat et al [3], and Park et al [4] have shown that video-assisted instructions are superior to verbal or standard paper instructions for cleanout quality.

Studies by Lorenzo-Zúñiga et al [5] and Kang et al [6] have shown that instructions delivered via interactive smartphone apps provide superior preparation for adult patients undergoing colonoscopy prep. Health care apps have been successfully used in other fields of medicine, including apps for asthma control [7] and smoking cessation [8].

We created a smartphone app (*SB Colonoscopy Prep*) that informs patients about their colonoscopy procedure, alerts them when to take their medications throughout the hours-long colonoscopy prep process, and tells them when to arrive at the endoscopy suite. We designed a study to determine if this app will yield improved colonoscopy cleanouts, better patient understanding of the procedure, fewer calls to the gastroenterology (GI) clinic, and more punctual arrival times to the endoscopy suite compared to patients who receive noninteractive written instructions.

## Methods

In total, 46 patients aged 5-18 years scheduled to undergo a diagnostic and/or therapeutic colonoscopy were recruited for the study. Subjects were volunteers recruited from Stony Brook Children’s Hospital’s pediatric GI service who were already scheduled to have a colonoscopy.

Exclusion criteria included patients who had undergone a colonoscopy within the past 1 year, patients admitted for a nasogastric cleanout, patients requiring colonoscopy preparation medication other than polyethylene glycol, or patients with a poor understanding of English. Subjects who had undergone a colonoscopy within the previous year were excluded as their prep quality may be influenced by information gathered from their previous procedure rather than the instructions provided to them in our study. Ownership of a smartphone was not required for inclusion in this study. A device was made available for subjects if they did not have a smartphone.

With a grant provided by NASPGHAN (North American Society for Pediatric Gastroenterology, Hepatology and Nutrition), we developed, to our knowledge, the first pediatric-focused smartphone app (that is also compatible for use on a tablet) aimed to help children and their parents prepare for a colonoscopy. The app delivers the same content as the written colonoscopy preparation instructions previously given to patients at our institution but utilizes the advantages inherent to an interactive, multimedia, and patient-operated app.

Subjects were assigned via block randomization to receive either app-based (intervention) or written (control) prep instructions. Subjects in the control group were given a 3-page document that described the procedure and instructed users on how to take the preparation medications. Both groups were provided with an identical list of frequently asked questions about colonoscopies. A link to a website where users could view an animated video was included in the written instructions. The written instructions also contained the time and date of the procedure. All subjects were instructed to arrive 1 hour before their scheduled procedure.

For their colonoscopy prep, patients in both the intervention and control groups were instructed to first take bisacodyl at a specified dosage. Following the bisacodyl, they took a specified amount of polyethylene glycol and mixed it into a specified amount of liquid. Patients drank half of the mixture over 4 hours, took a 2-hour break, and then finished the second half of the prep over the course of another 4 hours. Specific dosages of bisacodyl and polyethylene glycol were based on the patient’s weight.

**Figure 1:**
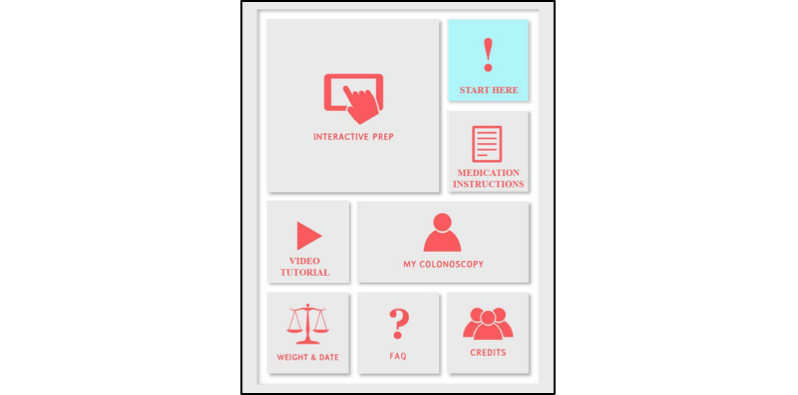
The home screen of the *SB Colonoscopy Prep* app.

**Figure 2:**
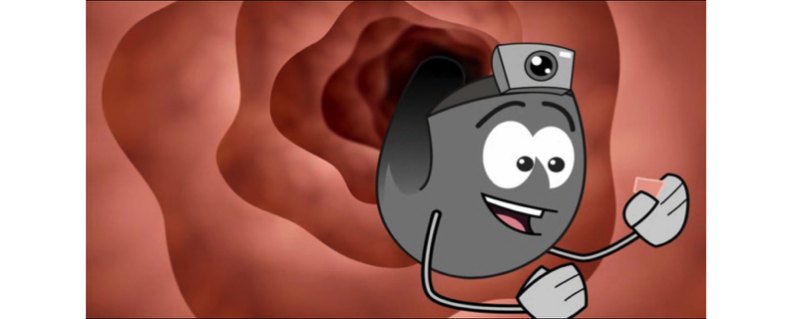
The animated colonoscope “Scopey” featured in the app’s video.

Patients in the intervention group downloaded a free app from the iOS App or Google Play stores called *SB Colonoscopy Prep*. Upon opening the app, users were directed to a home screen containing 8 icons (Figure 1). Users began by clicking the “Start Here!” icon, introducing them to the app and its functions. “Medication Instructions” contains the same instructions the control users received via paper format. “Interactive Prep” walks the patient through the colonoscopy prep process in real time, reminding them what medications to take and when to take them. Subjects received either an audible or vibrating alarm on their smartphones alerting them when it was time to take a new medication, when to take a 2-hour break, and when their prep was complete. For simplicity, the app only provides instructions pertaining to the most commonly used colonoscopy preparation medications at our institution (ie, bisacodyl and polyethylene glycol). “Video Tutorial” contains a short, animated video of a young boy walking the patient through the colonoscopy process using easy-to-understand vocabulary (Multimedia Appendix 1). The boy explains what a colonoscopy is, why prep is important, and what patients can expect when they arrive at the colonoscopy suite. The video also features an animated, smiling colonoscope named “Scopey” designed to be a lighthearted but accurate portrayal of the colonoscopy procedure itself (Figure 2). The video was written and directed by authors DL and BD and animated by CI Design.

“My Colonoscopy” gives users the time and date of their procedure as well as instructions to arrive 1 hour prior to their procedure. Patients are provided with driving, walking. or public transportation directions to our institution. Directions were generated using Google Maps and the patient’s current GPS location. Before generating directions, the smartphone asked subjects to grant permission to allow the app to access the user’s current location. “Weight & Date” allows users to enter their weight and time and date of their colonoscopy. “FAQs” provides a list of frequently asked questions. Lastly, “Credits” lists the people and institutions responsible for creating the app.

Prep quality was measured with the validated Boston Scoring Scale [9]. A score of 0, 1, 2, or 3 is given to the right, transverse, and left colon based on the amount and consistency of stool visualized as well as the ease or difficulty of guiding endoscopic instrumentation during a colonoscopy. Higher scores indicate a cleaner colon and a Boston score of 7 or above indicates an “excellent” prep. To eliminate bias, the 4 grading gastroenterologists in our study did not know whether subjects had used written or app instructions for their preps.

Patient arrival time at the hospital was taken from their electronic medical record as the time the patient signed in at the endoscopy suite. The number of telephone calls to the GI service from subjects was recorded.

A written questionnaire was given to subjects’ parents on the day of the colonoscopy using validated questions from in two previous studies on colonoscopy prep [10,11]. In the questionnaire, parents were asked to grade their knowledge about the procedure both before and after receiving either the app or written information. The patients were allowed to participate in the survey with their parents.

Of the 46 patients recruited, 23 were assigned to receive app instructions and 23 were assigned to receive traditional paper-based instructions. In total, 42 patients completed the study. Three subjects withdrew from the study. Two subjects forgot about their enrollment in the study and called our service for paper-based instructions. One subject who has been randomly assigned to receive app-based instructions became nervous about using the app and decided against using an experimental prep process. One control subject consented but was withdrawn after his prep was changed to another laxative formulation. Our final subject sample comprised 22 control and 20 experimental subjects.Statistical analysis was performed using IBM SPSS Statistics, version 24.0 (IBM Corporation).

The study was approved by the Stony Brook University Hospital Institutional Review Board (#1132702) on November 15, 2014. All study data were collected between July 15, 2015, and May 1, 2020.

## Results

App users had a mean Boston Scoring Scale score of 7.2 (range 3-9) versus a mean score of 5.9 (range 3-9) for users with written instruction (*P*=.02) (Table 1). In the app group, 75% (15/20) of users’ Boston scores were 7 or above and therefore labeled as “excellent” preps. In the control group, 41% (9/22) of written users’ Boston scores were categorized as “excellent” with scores of 7 or above (Table 2). On average, app users arrived 46 minutes and control users arrived 44 minutes prior to their procedure, with no significant difference between the two groups (*P*=.56). Based on questionnaire results obtained from subjects, 50% (10/20) of app users had improved knowledge of the colonoscopy versus 36.4% (8/22) of control subjects (*P*=.37).

All 20 app users were able to download, install, and operate the software without technical difficulties. Based on their survey answers, all app users completed the prep within the recommended time whereas only 18 patients (81.8%) in the control group managed to do this (*P*=.45). In total, 6 phone calls were made to the GI service by controls versus 2 calls from app users (*P*=.27).

**Table 1:** Study results.

Variable	Control subjects (written, n=22)	Experimental subjects (app, n=20)	*P* value
Age range (years)	6-18	5-18	—^a^
Average Boston score	5.9	7.2	.02
Patient arrival time to endoscopy suite (minutes before procedure)	44	46	.56
Calls to gastroenterology service, n	2	6	.27
Subjects with improved knowledge after receiving materials, n (%)	9 (36)	10 (50)	.37

^a^Not applicable

**Table 2:** Boston scores for app and written instruction users.

Boston score	Subjects	
	Written (n=22), n (%)	App (n=20), n (%)
3	1 (5)	1 (5)
4	5 (23)	0 (0)
5	5 (23)	2 (10)
6	2 (9)	2 (10)
7	4 (18)	6 (30)
8	3 (14)	4 (20)
9	2 (9)	5 (25)

## Discussion

### Principal Findings

Subjects who used an app to guide them through their colonoscopy prep had better prepped colons compared with patients who used paper-based instructions for their prep. App users had higher Boston scores, indicating better preps, and a higher percentage of scores classified as “excellent cleanout” than patients who used written instructions. This is similar to studies in adults comparing colonoscopy prep quality in subjects using app versus paper-based instructions. In a study by Lorenzo-Zúñiga et al [5], 100% (N=108) of the subjects using an interactive app for colonoscopy prep had a successful bowel prep compared to 96.1% (146/152) of those in the control group receiving written instructions for their preps (*P*=.037). Kang et al [6] demonstrated the benefits of app-based instructions, with 82.2% (318/387) of preps deemed adequate in adult subjects who used an interactive app vs 69.5% (266/383) of preps deemed adequate in control subjects who received written instructions (*P*=.001).

While both groups in our study received the same instructions about colonoscopy prep, including medication names, dosages, and when to take each medication, the app had features that reduced the chances of missed or delayed medications. The app users had this information continually given to them throughout the prep process. For example, 4 hours after app users began to take the polyethylene glycol mixture, the app alerted them that it was time to take a 2-hour break. Two hours later, the app alerted users to begin the second half of the prep. Sixty minutes before the patient was supposed to finish their prep, the app alerted users that they had 1 hour left to finish taking their medications. There was a countdown clock visible to the user throughout the prep process, informing them how much time was left in each part of the prep process. Even when their smartphone display screens were off, the app caused the smartphone to sound an audible alarm or vibrate and flash information on the screen about the next step in the prep process.

### Limitations

Patient knowledge, the number of phone calls to the GI clinic, and punctuality did not show statistical differences between the 2 study groups. This may be due to the small number of subjects in the study. Future studies with a larger sample size may be needed to increase study power to investigate if an interactive app could affect these variables.

The use of a mobile phone–based health aid does not always result in improved outcomes, and the availability of such apps does not mean patients will want to use them. A study by Ting et al [12] on 70 adolescents with systemic lupus erythematous failed to show improvement with their hydroxychloroquine adherence while using a mobile phone–based reminder system. Perski et al [13] demonstrated that nonmedical factors such as the color scheme, design, and user ratings influence whether users will choose to use an app [13]. There is still insufficient economic data supporting the use of mobile health apps. Many smartphone app studies to date have been pilot studies with limited data [14]. Reviews of these studies also suggest there may be a lack of data on the benefits of smartphone-based health apps in low-income communities [15].

### Conclusions

This is the first study to demonstrate significant improvement in colonoscopy prep in pediatric patients with the use of a smartphone or tablet app in a typical clinical setting. Future studies will continue to investigate the benefits of mobile health software in the management of pediatric patients and their families.

## Appendix

Multimedia Appendix 1 “Upper and Lower Colonoscopy” video by the Children’s Hospital of Wisconsin.

Multimedia Appendix 2 CONSORT-eHEALTH Checklist (V1.6.2).

## Abbreviations

GI: gastroenterology
NASPGHAN: North American Society for Pediatric Gastroenterology, Hepatology and Nutrition
