# Supplementary material for: The Impact of a Smartphone App on the Quality of Pediatric Colonoscopy Preparations: Randomized Controlled Trial
Source: JMIR Pediatr Parent. 2020 Nov 10;3(2):e18174. doi: 10.2196/18174 (PMC7685924; doi:10.2196/18174)
Supplement: Multimedia Appendix 2 [file pediatrics_v3i2e18174_app2.pdf]

CONSORT-EHEALTH Checklist V1.6.2 Report	Manuscript Number	18174

(based on CONSORT-EHEALTH V1.6), available at [http://tinyurl.com/consort-ehealth-v1-6].

Date completed

6/30/2020 11:01:22

by

James Brief

The Impact of a Novel Smartphone App on the Quality of Pediatric Colonoscopy Preparations: Randomized Controlled Trial

TITLE

1a-i) Identify the mode of delivery in the title

The Impact of a "Novel Smartphone App" on the Quality of Pediatric Colonoscopy Preparations: Randomized Controlled Trial

1a-ii) Non-web-based components or important co-interventions in title

1a-iii) Primary condition or target group in the title

The Impact of a Novel Smartphone App on the Quality of "Pediatric Colonoscopy Preparations:" Randomized Controlled Trial

ABSTRACT

1b-i) Key features/functionalities/components of the intervention and comparator in the METHODS section of the ABSTRACT

We state that In total, 46 patients aged 5-18 years "received either app-based or written material with instructions on how to take their prep medications as well as information about the colonoscopy procedure." Prep quality, number of calls to the gastroenterology service, and patient arrival time were recorded. After the procedure, a questionnaire was given to each patient in which they graded their knowledge of the procedure both before and after receiving the app or written material.

1b-ii) Level of human involvement in the METHODS section of the ABSTRACT

subjects involved were "46 patients aged 5-18 years"

1b-iii) Open vs. closed, web-based (self-assessment) vs. face-to-face assessments in the METHODS section of the ABSTRACT

Subjects were "46 patients aged 5-18 years." We defined patients in the paper as children already scheduled to undergo a colonoscopy

1b-iv) RESULTS section in abstract must contain use data

We state that "App users had higher mean Boston scores versus control subjects receiving written instructions (7.2 vs 5.9, P=.02), indicating better colonoscopy preps. In total, 75% (15/20) of app users and 41% (9/22) of written instruction users had preps categorized as “excellent” on the Boston scale. We found no significant differences in knowledge about the procedure (app users: 10/20 [50%], written instruction users 8/21 [36%]; P=.37), phone calls to the gastroenterology clinic (n=6 vs n=2; P=.27), or arrival times at the endoscopy suite (44 min vs 46 min before the scheduled procedure time; P=.56)."

1b-v) CONCLUSIONS/DISCUSSION in abstract for negative trials

We define both the benefits of the app as well as those aspects that were not affected: "Smartphone app use was associated with an increased number of colonoscopy preps classified as “excellent” on the Boston scale. There was no significant difference between app users and the control group regarding the number of calls to the gastroenterology clinic, patient arrival time, or patient knowledge about the procedure."

INTRODUCTION

2a-i) Problem and the type of system/solution

The problem is that "Up to one-third of children undergoing [colonoscopies] are reported to have suboptimal colon preparation."We decided to see if our app-based material would provide benefits over written/paper-based materials for the pediatric population and their families helping them prep.

2a-ii) Scientific background, rationale: What is known about the (type of) system

There are already studies on the matter of apps benefitting healthcare. "Studies by Lorenzo-Zúñiga et al and Kang et al have shown that instructions delivered via interactive smartphone apps provide superior preparation for adult patients undergoing colonoscopy prep. Health care apps have been successfully used in other fields of medicine, including apps for asthma control and smoking cessation."

Does your paper address CONSORT subitem 2b?

"We created a smartphone app that informs patients about their colonoscopy procedure, alerts them when to take their medications throughout the hours-long colonoscopy prep process, and tells them when to arrive at the endoscopy suite. We designed a study to determine if this app will yield improved colonoscopy cleanouts, better patient understanding of the procedure, fewer calls to the gastroenterology (GI) clinic, and more punctual arrival times to the endoscopy suite compared to patients who receive noninteractive written instructions."

METHODS

3a) CONSORT: Description of trial design (such as parallel, factorial) including allocation ratio

"Subjects were assigned via block randomization to receive either app-based (intervention) or written (control) prep instructions. Subjects in the control group were given a 3-page document that described the procedure and instructed users on how to take the preparation medications. Both groups were provided with an identical list of frequently asked questions about colonoscopies. A link to a website where users could view the animated video was included in the written instructions. The written instructions also contained the time and date of the procedure. All subjects were instructed to arrive 1 hour before their scheduled procedure."

3b) CONSORT: Important changes to methods after trial commencement (such as eligibility criteria), with reasons

"Subjects were volunteers recruited from Stony Brook Children’s Hospital’s pediatric GI service who were already scheduled to have a colonoscopy. Exclusion criteria included patients who had undergone a colonoscopy within the past 1 year, patients admitted for a nasogastric cleanout, patients requiring colonoscopy preparation medication other than polyethylene glycol. or patients with a poor understanding of English. Subjects who had undergone a colonoscopy within the previous year were excluded as their prep quality might be secondary to information gathered from their previous procedure rather than the instructions provided to them in our study. Ownership of a smartphone was not required for inclusion into this study. A device was made available for subjects if they did not have a smartphone. "

3b-i) Bug fixes, Downtimes, Content Changes

No. We addressed the difficulty of bug fixes in a previously submitted draft but this was cut down in the final paper to narrow the focus to the medical aspects. The editor also requested the submitted draft be shortened for consideration of publication.

4a) CONSORT: Eligibility criteria for participants

"Subjects were volunteers recruited from Stony Brook Children’s Hospital’s pediatric GI service who were already scheduled to have a colonoscopy."

4a-i) Computer / Internet literacy

"Ownership of a smartphone was not required for inclusion into this study. A device was made available for subjects if they did not have a smartphone. "

4a-ii) Open vs. closed, web-based vs. face-to-face assessments:

"Subjects were volunteers recruited from Stony Brook Children’s Hospital’s pediatric GI service who were already scheduled to have a colonoscopy."

4a-iii) Information giving during recruitment

Subjects were recruited AFTER it was decided that they needed a colonoscopy. "Subjects were volunteers recruited from Stony Brook Children’s Hospital’s pediatric GI service who were already scheduled to have a colonoscopy."

4b) CONSORT: Settings and locations where the data were collected

All subjects were recruited in Stony Brook, New York, USA. "Subjects were volunteers recruited from Stony Brook Children’s Hospital’s pediatric GI service who were already scheduled to have a colonoscopy."

4b-i) Report if outcomes were (self-)assessed through online questionnaires

We used written questionnaires, not online ones. "A written questionnaire was given to subjects’ parents on the day of the colonoscopy"

4b-ii) Report how institutional affiliations are displayed

"Subjects were volunteers recruited from Stony Brook Children’s Hospital’s pediatric GI service who were already scheduled to have a colonoscopy."
Multimedia appendix says “Upper and Lower Colonoscopy video by the Children’s Hospital of Wisconsin."

5) CONSORT: Describe the interventions for each group with sufficient details to allow replication, including how and when they were actually administered

5-i) Mention names, credential, affiliations of the developers, sponsors, and owners

As stated in the manuscript, "a grant [was] provided by NASPGHAN (North American Society for Pediatric Gastroenterology, Hepatology and Nutrition)."

Conflict of Interest Disclosed: Diana Lerner (co-author of manuscript) is a co-founder of Lerner Media Inc. This company helps with multimedia production.

5-ii) Describe the history/development process

5-iii) Revisions and updating

5-iv) Quality assurance methods

5-v) Ensure replicability by publishing the source code, and/or providing screenshots/screen-capture video, and/or providing flowcharts of the algorithms used

**5-vi) Digital preservation**		
**5-vii) Access**		
The app was free and ownership of a phone was not required for the study. "Patients in the intervention group downloaded a free app." and "Ownership of a smartphone was not required for inclusion into this study. A device was made available for subjects if they did not have a smartphone. "		
**5-viii) Mode of delivery, features/functionalities/components of the intervention and comparator, and the theoretical framework**		
"Directions were generated using Google Maps and the patient's current GPS location. "		
**5-ix) Describe use parameters**		
Subjects were to use the app on the day before their colonoscopy. "For their colonoscopy prep, patients in both intervention and control groups were instructed to first take bisacodyl at a specified dosage [using either the app or the written instructions]"		
**5-x) Clarify the level of human involvement**		
**5-xi) Report any prompts/reminders used**		
"The app caused the smartphone to sound an audible alarm or vibrate and flash information on the screen about the next step in the prep process"		
**5-xii) Describe any co-interventions (incl. training/support)**		
Patients were able to call the Gastroenterology service if they had questions. "The number of telephone calls to the GI service from subjects was recorded... In total, 6 phone calls were made to the GI service by controls versus 2 calls from app users"		
**6a) CONSORT: Completely defined pre-specified primary and secondary outcome measures, including how and when they were assessed**		
"Subjects who used an app to guide them through their colonoscopy prep had better prepped colons compared with patients who used paper-based instructions for their prep. App users had higher Boston scores, indicating better preps, and a higher percentage of scores classified as "excellent cleanout" than patients who used written instructions[...] Patient knowledge, the number of phone calls to the GI clinic, and punctuality did not show statistical differences between the 2 study groups."		
**6a-i) Online questionnaires: describe if they were validated for online use and apply CHERRIES items to describe how the questionnaires were designed/deployed**		
**6a-ii) Describe whether and how "use" (including intensity of use/dosage) was defined/measured/monitored**		
**6a-iii) Describe whether, how, and when qualitative feedback from participants was obtained**		
**6b) CONSORT: Any changes to trial outcomes after the trial commenced, with reasons**		
All subjects were recruited in Stony Brook, New York, USA. "Subjects were volunteers recruited from Stony Brook Children's Hospital's pediatric GI service who were already scheduled to have a colonoscopy."		
**7a) CONSORT: How sample size was determined**		
**7a-i) Describe whether and how expected attrition was taken into account when calculating the sample size**		
**7b) CONSORT: When applicable, explanation of any interim analyses and stopping guidelines**		
"Subjects who used an app to guide them through their colonoscopy prep had better prepped colons compared with patients who used paper-based instructions for their prep. App users had higher Boston scores, indicating better preps, and a higher percentage of scores classified as "excellent cleanout" than patients who used written instructions[...] Patient knowledge, the number of phone calls to the GI clinic, and punctuality did not show statistical differences between the 2 study groups."		
**8a) CONSORT: Method used to generate the random allocation sequence**		
"To eliminate bias, the 4 grading gastroenterologists in our study did not know whether subjects had used written or app instructions for their preps. "		
**8b) CONSORT: Type of randomisation; details of any restriction (such as blocking and block size)**		
"Subjects were assigned via block randomization to receive either app-based (intervention) or written (control) prep instructions."		
**9) CONSORT: Mechanism used to implement the random allocation sequence (such as sequentially numbered containers), describing any steps taken to conceal the sequence until interventions were assigned**		
"Subjects were assigned via block randomization to receive either app-based (intervention) or written (control) prep instructions."		
**10) CONSORT: Who generated the random allocation sequence, who enrolled participants, and who assigned participants to interventions**		
"Subjects were assigned via block randomization to receive either app-based (intervention) or written (control) prep instructions."		
**11a) CONSORT: Blinding - If done, who was blinded after assignment to interventions (for example, participants, care providers, those assessing outcomes) and how**		
**11a-i) Specify who was blinded, and who wasn't**		
"To eliminate bias, the 4 grading gastroenterologists in our study did not know whether subjects had used written or app instructions for their preps. "		
**11a-ii) Discuss e.g., whether participants knew which intervention was the "intervention of interest" and which one was the "comparator"**		
**11b) CONSORT: If relevant, description of the similarity of interventions**		
We strived to make sure both groups received the same information in different forms (app versus written materials). "Subjects in the control group were given a 3-page document that described the procedure and instructed users on how to take the preparation medications. Both groups were provided with an identical list of frequently asked questions about colonoscopies. A link to a website where users could view the animated video was included in the written instructions. The written instructions also contained the time and date of the procedure. All subjects were instructed to arrive 1 hour before their scheduled procedure."		
**12a) CONSORT: Statistical methods used to compare groups for primary and secondary outcomes**		
"Statistical analysis was performed using IBM SPSS Statistics, version 24.0 (IBM Corporation). "		
**12a-i) Imputation techniques to deal with attrition / missing values**		
"Three subjects withdrew from the study. Two subjects forgot about their enrollment in the study and called our service for paper-based instructions. One subject who has been randomly assigned to receive app-based instructions became nervous about using the app and decided against using an experimental prep process. One control subject consented but was withdrawn after his prep was changed to another laxative formulation. Our final subject sample comprised 22 control and 20 experimental subjects. "		
**12b) CONSORT: Methods for additional analyses, such as subgroup analyses and adjusted analyses**		
Not applicable		
RESULTS		
**13a) CONSORT: For each group, the numbers of participants who were randomly assigned, received intended treatment, and were analysed for the primary outcome**		
1 center: "In total, 46 patients aged 5-18 years scheduled to undergo a diagnostic and/or therapeutic colonoscopy were recruited for the study. Subjects were volunteers recruited from Stony Brook Children's Hospital's pediatric GI service who were already scheduled to have a colonoscopy. " "Of the 46 patients recruited, 23 were assigned to receive app instructions and 23 were assigned to receive traditional paper-based instructions."		
**13b) CONSORT: For each group, losses and exclusions after randomisation, together with reasons**		
"Exclusion criteria included patients who had undergone a colonoscopy within the past 1 year, patients admitted for a nasogastric cleanout, patients requiring colonoscopy preparation medication other than polyethylene glycol. or patients with a poor understanding of English. Subjects who had undergone a colonoscopy within the previous year were excluded as their prep quality might be secondary to information gathered from their previous procedure rather than the instructions provided to them in our study. Ownership of a smartphone was not required for inclusion into this study. A device was made available for subjects if they did not have a smartphone. "		
**13b-i) Attrition diagram**		
**14a) CONSORT: Dates defining the periods of recruitment and follow-up**		
" All study data were collected between July 15, 2015, and May 1, 2020."		
**14a-i) Indicate if critical "secular events" fell into the study period**		
**14b) CONSORT: Why the trial ended or was stopped (early)**		
Trial was not stopped early. " All study data were collected between July 15, 2015, and May 1, 2020."		
**15) CONSORT: A table showing baseline demographic and clinical characteristics for each group**		
This is in Table 1.		
**15-i) Report demographics associated with digital divide issues**		
Provided in Table 1. Also "Ownership of a smartphone was not required for inclusion into this study. A device was made available for subjects if they did not have a smartphone. "		
**16a) CONSORT: For each group, number of participants (denominator) included in each analysis and whether the analysis was by original assigned groups**		
**16-i) Report multiple "denominators" and provide definitions**		

"Of the 46 patients recruited, 23 were assigned to receive app instructions and 23 were assigned to receive traditional paper-based instructions. In total, 42 patients completed the study. Three subjects withdrew from the study. Two subjects forgot about their enrollment in the study and called our service for paper-based instructions. One subject who has been randomly assigned to receive app-based instructions became nervous about using the app and decided against using an experimental prep process. One control subject consented but was withdrawn after his prep was changed to another laxative formulation. Our final subject sample comprised 22 control and 20 experimental subjects. "		
**16-ii) Primary analysis should be intent-to-treat**		
**17a) CONSORT: For each primary and secondary outcome, results for each group, and the estimated effect size and its precision (such as 95% confidence interval)**		
"App users had a mean Boston Scoring Scale score of 7.2 (range 3-9) versus a mean score of 5.9 (range 3-9) for users with written instruction (P=.02). In the app group, 75% (15/20) of users' Boston scores were 7 or above and therefore labeled as "excellent" preps. In the control group, 41% (9/22) of written users' Boston scores were categorized as "excellent" with scores of 7 or above (Figure 3). On average, app users arrived 46 minutes and control users arrived 44 minutes prior to their procedure, with no significant difference between the two groups (P=.56). Based on questionnaire results obtained from subjects, 50% (10/20) of app users had improved knowledge of the colonoscopy versus 36.4% (8/22) of control subjects (P=.37). "		
**17a-i) Presentation of process outcomes such as metrics of use and intensity of use**		
**17b) CONSORT: For binary outcomes, presentation of both absolute and relative effect sizes is recommended**		
I do not believe this is applicable		
**18) CONSORT: Results of any other analyses performed, including subgroup analyses and adjusted analyses, distinguishing pre-specified from exploratory**		
I do not believe this is applicable		
**18-i) Subgroup analysis of comparing only users**		
**19) CONSORT: All important harms or unintended effects in each group**		
I do not believe this is applicable. No "harms or unintended effects" occurred.		
**19-i) Include privacy breaches, technical problems**		
**19-ii) Include qualitative feedback from participants or observations from staff/researchers**		
DISCUSSION		
**20) CONSORT: Trial limitations, addressing sources of potential bias, imprecision, multiplicity of analyses**		
**20-i) Typical limitations in ehealth trials**		
"Patient knowledge, the number of phone calls to the GI clinic, and punctuality did not show statistical differences between the 2 study groups. This may be due to the small number of subjects in the study. Future studies with a larger sample size may be needed to increase study power to investigate if an interactive app could affect these variables. The use of a mobile phone–based health aid does not always result in improved outcomes, and the availability of such apps does not mean patients will want to use them."		
**21) CONSORT: Generalisability (external validity, applicability) of the trial findings**		
**21-i) Generalizability to other populations**		
**21-ii) Discuss if there were elements in the RCT that would be different in a routine application setting**		
**22) CONSORT: Interpretation consistent with results, balancing benefits and harms, and considering other relevant evidence**		
**22-i) Restate study questions and summarize the answers suggested by the data, starting with primary outcomes and process outcomes (use)**		
We state that "Subjects who used an app to guide them through their colonoscopy prep had better prepped colons compared with patients who used paper-based instructions for their prep. App users had higher Boston scores, indicating better preps, and a higher percentage of scores classified as "excellent cleanout" than patients who used written instructions. " We also state that "Patient knowledge, the number of phone calls to the GI clinic, and punctuality did not show statistical differences between the 2 study groups. "		
**22-ii) Highlight unanswered new questions, suggest future research**		
Other information		
**23) CONSORT: Registration number and name of trial registry**		
"The study was approved by the Stony Brook University Hospital Institutional Review Board on October 1, 2014. " Project (IRB) number 1132702.		
**24) CONSORT: Where the full trial protocol can be accessed, if available**		
"The study was approved by the Stony Brook University Hospital Institutional Review Board on October 1, 2014. " Project (IRB) number 1132702.		
**25) CONSORT: Sources of funding and other support (such as supply of drugs), role of funders**		
""The study was approved by the Stony Brook University Hospital Institutional Review Board on October 1, 2014. " Project (IRB) number 1132702."		
**X26-i) Comment on ethics committee approval**		
**x26-ii) Outline informed consent procedures**		
**X26-iii) Safety and security procedures**		
**X27-i) State the relation of the study team towards the system being evaluated**		
We disclose in the COI section that "Diana Lerner is a co-founder of Lerner Media Inc. This company helps with multimedia production."		
